# Di-*tert*-butyl (2*R*,3*R*)-2-{[(2*E*)-3-(4-acet­yloxy-3-meth­oxy­phen­yl)prop-2-eno­yl]­oxy}-3-hy­droxy­butane­dioate

**DOI:** 10.1107/S1600536812002784

**Published:** 2012-02-04

**Authors:** Josh L. Hixson, Dennis K. Taylor, Seik Weng Ng, Edward R. T. Tiekink

**Affiliations:** a School of Agriculture, Food and Wine, University of Adelaide, Waite Campus, PMB 1, Glen Osmond, SA 5064, Australia; b Department of Chemistry, University of Malaya, 50603 Kuala Lumpur, Malaysia; c Chemistry Department, Faculty of Science, King Abdulaziz University, PO Box 80203 Jeddah, Saudi Arabia

## Abstract

In the title mol­ecule, C_24_H_32_O_10_, one *tert*-butyl ester group is folded towards the central benzene ring while the other is directed away. The acetyl group is almost perpendicular to the benzene ring to which it is connected [C—C—O—C torsion angle = 90.4 (12)°]. The conformation about the ethene bond [1.313 (7) Å] is *E*. The atoms of the benzene ring and its attached ester group and part of the hy­droxy *tert*-butyl ester side chain are disordered over two sets of sites in a 50:50 ratio. Linear supra­molecular chains along the *a* axis mediated by hy­droxy–carbonyl O—H⋯O hydrogen bonds feature in the crystal packing. The same H atom also partakes in an intra­molecular O—H⋯O inter­action.

## Related literature

For background to the formation of the odorant 4-ethyl­guaiacol with relevance to the wine industry, see: Chatonnet *et al.* (1992 ▶); Hixson *et al.* (2012 ▶); Ong & Nagel (1978 ▶); Nagel & Wulf (1979 ▶); Zhao & Burke (1998 ▶). For the preparation and characterization of 1-*O*-acetyl ferulic acid; see: Zhao & Burke (1998 ▶); Hosoda *et al.* (2001 ▶).
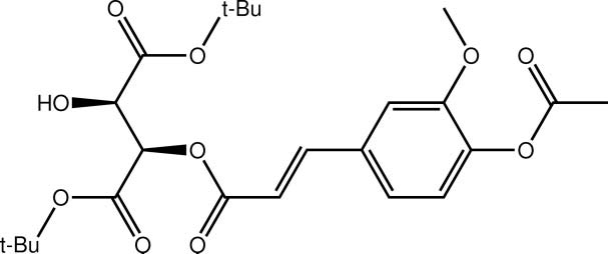


## Experimental

### 

#### Crystal data

C_24_H_32_O_10_
*M* *_r_* = 480.50
Monoclinic, 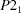

*a* = 5.9894 (1) Å
*b* = 10.6483 (1) Å
*c* = 19.6676 (2) Å
β = 96.324 (1)°
*V* = 1246.71 (3) Å^3^
*Z* = 2
Cu *K*α radiation
μ = 0.84 mm^−1^
*T* = 100 K
0.30 × 0.25 × 0.20 mm

#### Data collection

Agilent SuperNova Dual diffractometer with an Atlas detector
Absorption correction: multi-scan (*CrysAlis PRO*; Agilent, 2010 ▶) *T*_min_ = 0.736, *T*_max_ = 1.000
7492 measured reflections
4800 independent reflections
4762 reflections with *I* > 2σ(*I*)
*R*_int_ = 0.011

#### Refinement

*R*[*F* ^2^ > 2σ(*F* ^2^)] = 0.057
*wR*(*F* ^2^) = 0.159
*S* = 1.04
4800 reflections
326 parameters
113 restraints
H-atom parameters constrained
Δρ_max_ = 0.56 e Å^−3^
Δρ_min_ = −0.56 e Å^−3^
Absolute structure: Flack (1983 ▶), 2185 Friedel pairs
Flack parameter: 0.0 (2)

### 

Data collection: *CrysAlis PRO* (Agilent, 2010 ▶); cell refinement: *CrysAlis PRO*; data reduction: *CrysAlis PRO*; program(s) used to solve structure: *SHELXS97* (Sheldrick, 2008 ▶); program(s) used to refine structure: *SHELXL97* (Sheldrick, 2008 ▶); molecular graphics: *ORTEP-3* (Farrugia, 1997 ▶) and *DIAMOND* (Brandenburg, 2006 ▶); software used to prepare material for publication: *publCIF* (Westrip, 2010 ▶).

## Supplementary Material

Crystal structure: contains datablock(s) global, I. DOI: 10.1107/S1600536812002784/hb6612sup1.cif

Structure factors: contains datablock(s) I. DOI: 10.1107/S1600536812002784/hb6612Isup2.hkl

Supplementary material file. DOI: 10.1107/S1600536812002784/hb6612Isup3.cml

Additional supplementary materials:  crystallographic information; 3D view; checkCIF report

## Figures and Tables

**Table 1:** Hydrogen-bond geometry (Å, °)

*D*—H⋯*A*	*D*—H	H⋯*A*	*D*⋯*A*	*D*—H⋯*A*
O18—H18⋯O32	0.84	2.21	2.631 (8)	111
O18—H18⋯O22^i^	0.84	2.57	3.185 (5)	131

Symmetry code: (i) 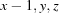.

**Fig. 1.**
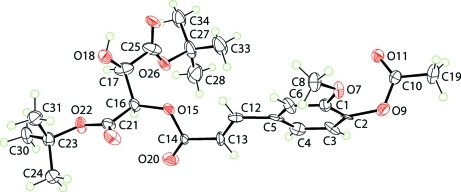
The molecular structure of the title compound showing the atom-labelling scheme and displacement ellipsoids at the 50% probability level. Only one component of the disordered atoms is shown for clarity.

**Fig. 2.**
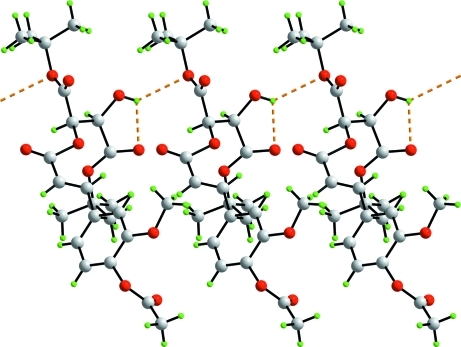
A view of the linear supramolecular chain along the a axis mediated by O—H···O hydrogen bonds shown as orange dashed lines. Only one component of the disordered atoms is shown for clarity.

## supplementary crystallographic information

### Comment

The breakdown of ferulic acid by *D. bruxellensis* to form the potent odorant 4-ethylguaiacol has been known for decades (Chatonnet *et al.*, 1992). Recently, it has been found that the metabolism of the ethyl ester of ferulic acid by this yeast can also result in the accumulation of 4-ethylguaiacol (Hixson *et al.*, 2012). Existing in both the grape berry and in wine in significant concentrations (Ong & Nagel, 1978; Nagel & Wulf, 1979), the feruloyl *L*-tartrate ester has the potential to contribute to the accumulation of 4-ethylguaiacol in finished wines and thus contribute further to the spoilage of wine. Synthesis of the known wine component feruloyl *L*-tartrate was achieved using di-*tert*-butyl *L*-tartrate and the 1-*O*-acetyl protected hydroxycinnamic acid in an analogous method to that described by Zhao & Burke (1998). The products of coupling were isolated and recrystallized from 30% ethyl acetate/hexane to afford a crystalline solid from which the structure was determined by X-ray crystallography to confirm the retention of the (*R*,*R*)-stereochemistry, Fig. 1.

The hydroxyl-H atom is bifurcated, forming an intramolecular O—H···O hydrogen bond with the adjacent carbonyl-O, Table, and an intermolecular O–H···O hydrogen bond with a translationally related carbonyl-O atom to form a linear supramolecular chain along the *a* axis, Fig. 1 and Table 1.

### Experimental

1-*O*-Acetyl ferulic acid was prepared using a method analogous to that previously described by Zhao and Burke (1998), and the characterization data matched that previously described (Hosoda *et al.*, 2001). 1-*O*-Acetyl ferulic acid (0.16 g, 0.67 mmol) was heated under reflux in dry benzene (10 ml) containing thionyl chloride (1 ml, 13.77 mmol). After 5 h the mixture was allowed to cool to room temperature and then concentrated *in vacuo*. The crude residue was taken up in dry benzene (3 ml) and added drop-wise to a solution of di-*tert*-butyl *L*-tartrate (0.21 g, 0.79 mmol) in dry pyridine (3 ml), then stirred at ambient temperature overnight. The mixture was concentrated and pyridine azeotropically removed with toluene. Purification with column chromatography (20% EtOAc/X4) and recrystallization from 30% EtOAc/X4 gave 154.0 mg (48%) of white crystals. *M*.pt 413.7–415.2 K. *R*_f_ (30% EtOAc/X4): 0.34 ^1^H NMR: (600 MHz, CDCl_3_) δ: 7.70 (d, 1H, J = 16.0 Hz, H_7_), 7.12–7.11 (m, 2H, H_3,5_), 7.05 (d, 1H, J = 8.6 Hz, H_6_), 6.45 (d, 1H, J = 16.0 Hz, H_8_), 5.50 (d, 1H, J = 2.3 Hz, H_2'_), 4.68 (dd, 1H, J = 6.8 and 2.3 Hz, H_3'_), 3.87 (s, 3H, OCH_3_), 3.21 (d, 1H, J = 6.8 Hz, OH), 2.33 (s, 3H, OCOCH_3_), 1.51 (s, 9H, *t*-Bu_4_), 1.44 (s, 9H, ^t^Bu_1_). ^13^C NMR: (600 MHz, CDCl_3_) δ: 170.2 (C_4'_), 168.9 (OCOCH_3_), 165.9 (C_1'_), 165.5 (C9), 151.5 (C7), 145.9 (C2), 141.8 (C1), 133.2 (C4), 123.4 (C6), 121.8 (C5), 116.8 (C8), 111.3 (C3), 84.1 (C_1_(CH_3_)_3_), 83.5 (C_4_(CH_3_)_3_), 73.5 (C_2'_), 71.0 (C_3'_), 56.1 (OMe), 28.1 (C_4_(CH_3_)_3_), 28.0 (C_1_(CH_3_)_3_), 20.8 (OCOCH_3_). Calc. C 59.99, H 6.71, O 33.30. Anal. C_24_H_32_O_10_ for C 59.79, H 6.73, O 33.48.

### Refinement

Carbon- and oxygen bound H-atoms were placed in calculated positions [C—H 0.95 to 1.00 and O—H 0.84 Å; *U*_iso_(H) 1.2 to 1.5*U*_eq_(C,*O*)] and were included in the refinement in the riding model approximation. The molecule is disordered in some parts of the molecule. The disorder was treated as a 1:1 type of disorder. The aromatic rings were refined as rigid hexagons of 1.39 Å sides. For the disordered atoms, pairs of 1,2-related bond distances were restrained to within 0.01 Å of each other; these included atom—atom_ordered_ as well as atom—atom_disordered_ distances. The displacement parameters of the primed atoms were set to those of the unprimed ones, and the anisotropic displacement factors of the disordered atom atoms were restrained to be nearly isotropic. The two *t*-butyl groups are both ordered. However, the vibration of one of the four-carbon groups had to be tightly restrained to be nearly isotropic.

### Crystal data

**Table:**

C_24_H_32_O_10_	*F*(000) = 512
*M**_r_* = 480.50	*D*_x_ = 1.280 Mg m^−^^3^
Monoclinic, *P*2_1_	Cu *K*α radiation, λ = 1.54184 Å
Hall symbol: P 2yb	Cell parameters from 5994 reflections
*a* = 5.9894 (1) Å	θ = 4.2–74.3°
*b* = 10.6483 (1) Å	µ = 0.84 mm^−^^1^
*c* = 19.6676 (2) Å	*T* = 100 K
β = 96.324 (1)°	Block, colourless
*V* = 1246.71 (3) Å^3^	0.30 × 0.25 × 0.20 mm
*Z* = 2	

### Data collection

**Table:**

Agilent SuperNova Dual diffractometer with an Atlas detector	4800 independent reflections
Radiation source: SuperNova (Cu) X-ray Source	4762 reflections with *I* > 2σ(*I*)
Mirror monochromator	*R*_int_ = 0.011
Detector resolution: 10.4041 pixels mm^-1^	θ_max_ = 74.5°, θ_min_ = 4.5°
ω scan	*h* = −7→7
Absorption correction: multi-scan (*CrysAlis PRO*; Agilent, 2010)	*k* = −13→12
*T*_min_ = 0.736, *T*_max_ = 1.000	*l* = −16→24
7492 measured reflections	

### Refinement

**Table:**

Refinement on *F*^2^	Hydrogen site location: inferred from neighbouring sites
Least-squares matrix: full	H-atom parameters constrained
*R*[*F*^2^ > 2σ(*F*^2^)] = 0.057	*w* = 1/[σ^2^(*F*_o_^2^) + (0.0976*P*)^2^ + 0.9408*P*] where *P* = (*F*_o_^2^ + 2*F*_c_^2^)/3
*wR*(*F*^2^) = 0.159	(Δ/σ)_max_ < 0.001
*S* = 1.04	Δρ_max_ = 0.56 e Å^−^^3^
4800 reflections	Δρ_min_ = −0.56 e Å^−^^3^
326 parameters	Extinction correction: *SHELXL97* (Sheldrick, 2008), Fc^*^=kFc[1+0.001xFc^2^λ^3^/sin(2θ)]^-1/4^
113 restraints	Extinction coefficient: 0.0092 (14)
Primary atom site location: structure-invariant direct methods	Absolute structure: Flack (1983), 2185 Friedel pairs
Secondary atom site location: difference Fourier map	Flack parameter: 0.0 (2)

### Special details

**Table:**

Geometry. All s.u.'s (except the s.u. in the dihedral angle between two l.s. planes) are estimated using the full covariance matrix. The cell s.u.'s are taken into account individually in the estimation of s.u.'s in distances, angles and torsion angles; correlations between s.u.'s in cell parameters are only used when they are defined by crystal symmetry. An approximate (isotropic) treatment of cell s.u.'s is used for estimating s.u.'s involving l.s. planes.
Refinement. Refinement of *F*^2^ against ALL reflections. The weighted *R*-factor *wR* and goodness of fit *S* are based on *F*^2^, conventional *R*-factors *R* are based on *F*, with *F* set to zero for negative *F*^2^. The threshold expression of *F*^2^ > 2σ(*F*^2^) is used only for calculating *R*-factors(gt) *etc*. and is not relevant to the choice of reflections for refinement. *R*-factors based on *F*^2^ are statistically about twice as large as those based on *F*, and *R*- factors based on ALL data will be even larger.

### Fractional atomic coordinates and isotropic or equivalent isotropic displacement parameters (Å^2^)

**Table:**

	*x*	*y*	*z*	*U*_iso_*/*U*_eq_	Occ. (<1)
O7	0.4371 (4)	0.5005 (2)	−0.08067 (10)	0.0278 (5)	
O9	0.6166 (4)	0.3370 (2)	−0.16084 (11)	0.0328 (5)	
O11	0.4029 (4)	0.1948 (2)	−0.11472 (11)	0.0292 (5)	
O22	1.3631 (4)	0.6050 (2)	0.41763 (10)	0.0310 (5)	
O26	1.0669 (4)	0.2356 (2)	0.32468 (11)	0.0347 (5)	
O29	1.2096 (4)	0.7128 (2)	0.32469 (12)	0.0397 (6)	
C8	0.3467 (6)	0.5990 (3)	−0.04224 (16)	0.0336 (7)	
H8A	0.2064	0.6296	−0.0671	0.050*	
H8B	0.4550	0.6681	−0.0359	0.050*	
H8C	0.3167	0.5667	0.0025	0.050*	
C10	0.4462 (5)	0.2526 (3)	−0.16343 (14)	0.0242 (6)	
C19	0.3237 (7)	0.2434 (4)	−0.23373 (16)	0.0424 (8)	
H19A	0.2985	0.1548	−0.2458	0.064*	
H19B	0.4135	0.2828	−0.2666	0.064*	
H19C	0.1788	0.2865	−0.2349	0.064*	
C16	1.1830 (5)	0.4859 (3)	0.33031 (14)	0.0276 (6)	
H16	1.3204	0.4323	0.3387	0.033*	0.50
H16'	1.3135	0.4291	0.3258	0.033*	0.50
C17	1.0120 (6)	0.4317 (3)	0.3743 (2)	0.0398 (8)	
H17	1.1049	0.4023	0.4167	0.048*	0.50
H17'	1.0675	0.4238	0.4240	0.048*	0.50
C21	1.2513 (5)	0.6166 (3)	0.35547 (15)	0.0281 (6)	
C23	1.4332 (6)	0.7160 (3)	0.46048 (15)	0.0288 (6)	
C24	1.5891 (5)	0.7998 (3)	0.42441 (15)	0.0307 (6)	
H24A	1.5054	0.8372	0.3838	0.046*	
H24B	1.7137	0.7496	0.4107	0.046*	
H24C	1.6486	0.8666	0.4556	0.046*	
C27	1.0144 (9)	0.1107 (4)	0.29275 (19)	0.0545 (11)	
C28	1.2436 (10)	0.0608 (4)	0.2822 (3)	0.0677 (13)	
H28A	1.3319	0.0486	0.3266	0.102*	
H28B	1.3203	0.1210	0.2550	0.102*	
H28C	1.2275	−0.0197	0.2579	0.102*	
C30	1.5590 (6)	0.6561 (3)	0.52404 (16)	0.0350 (7)	
H30A	1.6931	0.6130	0.5115	0.052*	
H30B	1.4609	0.5953	0.5435	0.052*	
H30C	1.6036	0.7214	0.5579	0.052*	
C31	1.2248 (6)	0.7844 (3)	0.47798 (18)	0.0377 (7)	
H31A	1.1468	0.8215	0.4364	0.057*	
H31B	1.2684	0.8509	0.5112	0.057*	
H31C	1.1248	0.7249	0.4976	0.057*	
C33	0.8689 (11)	0.1311 (6)	0.2251 (2)	0.0820 (18)	
H33A	0.9517	0.1817	0.1946	0.123*	
H33B	0.7308	0.1751	0.2335	0.123*	
H33C	0.8306	0.0497	0.2037	0.123*	
C34	0.9014 (10)	0.0297 (4)	0.3424 (2)	0.0612 (12)	
H34A	1.0038	0.0178	0.3843	0.092*	
H34B	0.8635	−0.0521	0.3214	0.092*	
H34C	0.7640	0.0712	0.3536	0.092*	
C25	0.9092 (6)	0.3113 (4)	0.34205 (19)	0.0426 (9)	
O15	1.1121 (8)	0.4798 (6)	0.2571 (3)	0.0228 (10)	0.50
O18	0.8662 (8)	0.5278 (4)	0.3987 (3)	0.0307 (7)	0.50
H18	0.7365	0.4983	0.3996	0.046*	0.50
O20	1.4905 (9)	0.4773 (6)	0.2372 (3)	0.0428 (11)	0.50
O32	0.7017 (9)	0.3148 (6)	0.3472 (3)	0.0330 (11)	0.50
C5	0.9150 (8)	0.4130 (9)	0.0399 (2)	0.0257 (15)	0.50
C6	0.7206 (15)	0.4724 (12)	0.0112 (5)	0.0288 (12)	0.50
H6	0.6447	0.5298	0.0376	0.035*	0.50
C1	0.637 (2)	0.4479 (19)	−0.0563 (5)	0.0238 (10)	0.50
C2	0.748 (2)	0.3639 (18)	−0.0950 (4)	0.0208 (17)	0.50
C3	0.9427 (17)	0.3045 (11)	−0.0662 (4)	0.0312 (15)	0.50
H3	1.0185	0.2471	−0.0926	0.037*	0.50
C4	1.0261 (11)	0.3290 (9)	0.0013 (3)	0.0329 (17)	0.50
H4	1.1590	0.2884	0.0209	0.039*	0.50
C12	0.9996 (9)	0.4465 (5)	0.1183 (3)	0.0235 (9)	0.50
H12	0.8911	0.4701	0.1477	0.028*	0.50
C13	1.2118 (9)	0.4426 (6)	0.1434 (3)	0.0247 (8)	0.50
H13	1.3169	0.4233	0.1121	0.030*	0.50
C14	1.3058 (13)	0.4652 (7)	0.2160 (3)	0.0211 (10)	0.50
O15'	1.0409 (8)	0.5056 (6)	0.2672 (3)	0.0228 (10)	0.50
O18'	0.8121 (7)	0.5086 (4)	0.3612 (3)	0.0307 (7)	0.50
H18'	0.6982	0.4653	0.3667	0.046*	0.50
O20'	1.3768 (11)	0.4694 (8)	0.2179 (4)	0.0428 (11)	0.50
O32'	0.7143 (9)	0.2721 (6)	0.3262 (3)	0.0330 (11)	0.50
C5'	0.9726 (8)	0.4098 (9)	0.0235 (2)	0.0257 (15)	0.50
C6'	0.7657 (16)	0.4690 (12)	0.0099 (5)	0.0288 (12)	0.50
H6'	0.7173	0.5273	0.0418	0.035*	0.50
C1'	0.6298 (18)	0.4431 (19)	−0.0504 (6)	0.0238 (10)	0.50
C2'	0.7007 (19)	0.3579 (18)	−0.0971 (5)	0.0208 (17)	0.50
C3'	0.9076 (17)	0.2987 (11)	−0.0835 (4)	0.0312 (15)	0.50
H3'	0.9561	0.2405	−0.1154	0.037*	0.50
C4'	1.0436 (11)	0.3246 (9)	−0.0232 (3)	0.0329 (17)	0.50
H4'	1.1850	0.2841	−0.0139	0.039*	0.50
C12'	1.0951 (9)	0.4272 (5)	0.0934 (3)	0.0235 (9)	0.50
H12'	1.2474	0.4006	0.0988	0.028*	0.50
C13'	1.0193 (11)	0.4750 (6)	0.1491 (3)	0.0247 (8)	0.50
H13'	0.8696	0.5049	0.1482	0.030*	0.50
C14'	1.1755 (11)	0.4797 (6)	0.2121 (3)	0.0211 (10)	0.50

### Atomic displacement parameters (Å^2^)

**Table:**

	*U*^11^	*U*^22^	*U*^33^	*U*^12^	*U*^13^	*U*^23^
O7	0.0346 (10)	0.0278 (10)	0.0191 (9)	0.0055 (9)	−0.0054 (7)	−0.0043 (8)
O9	0.0478 (12)	0.0274 (11)	0.0254 (10)	−0.0106 (10)	0.0145 (9)	−0.0049 (8)
O11	0.0318 (10)	0.0317 (11)	0.0233 (10)	−0.0058 (9)	−0.0012 (8)	0.0043 (9)
O22	0.0488 (12)	0.0215 (10)	0.0205 (10)	−0.0008 (9)	−0.0068 (8)	0.0014 (8)
O26	0.0441 (13)	0.0264 (11)	0.0310 (11)	−0.0172 (9)	−0.0076 (9)	0.0017 (9)
O29	0.0428 (13)	0.0344 (13)	0.0372 (12)	−0.0119 (10)	−0.0169 (10)	0.0191 (10)
C8	0.0429 (17)	0.0298 (16)	0.0265 (14)	0.0091 (14)	−0.0031 (12)	−0.0054 (12)
C10	0.0298 (14)	0.0209 (13)	0.0223 (13)	0.0043 (11)	0.0047 (10)	−0.0010 (11)
C19	0.069 (2)	0.0356 (18)	0.0203 (14)	0.0014 (17)	−0.0026 (14)	0.0006 (13)
C16	0.0322 (14)	0.0292 (15)	0.0193 (13)	−0.0102 (12)	−0.0072 (11)	0.0050 (11)
C17	0.0406 (18)	0.0251 (16)	0.056 (2)	0.0031 (14)	0.0179 (15)	0.0153 (15)
C21	0.0253 (13)	0.0311 (15)	0.0262 (14)	−0.0063 (12)	−0.0051 (10)	0.0077 (13)
C23	0.0421 (16)	0.0189 (13)	0.0248 (14)	−0.0024 (12)	0.0000 (11)	0.0002 (11)
C24	0.0353 (15)	0.0263 (15)	0.0299 (15)	0.0005 (12)	0.0019 (11)	0.0065 (12)
C27	0.092 (3)	0.0381 (18)	0.0305 (17)	−0.038 (2)	−0.0078 (18)	−0.0010 (15)
C28	0.111 (4)	0.040 (2)	0.056 (2)	−0.019 (2)	0.025 (2)	−0.0203 (19)
C30	0.056 (2)	0.0237 (14)	0.0226 (15)	−0.0055 (14)	−0.0075 (13)	0.0000 (12)
C31	0.0429 (17)	0.0308 (17)	0.0406 (18)	−0.0026 (14)	0.0105 (14)	−0.0017 (14)
C33	0.121 (4)	0.083 (3)	0.036 (2)	−0.070 (3)	−0.017 (2)	0.015 (2)
C34	0.099 (3)	0.047 (2)	0.0349 (18)	−0.040 (2)	−0.0053 (18)	0.0038 (16)
C25	0.0330 (16)	0.045 (2)	0.0472 (19)	−0.0140 (15)	−0.0096 (13)	0.0300 (17)
O15	0.019 (3)	0.027 (3)	0.0207 (17)	−0.0050 (19)	−0.0049 (17)	0.0026 (15)
O18	0.0330 (17)	0.0225 (15)	0.036 (2)	−0.0023 (13)	0.0025 (16)	−0.0022 (16)
O20	0.025 (2)	0.063 (2)	0.039 (2)	−0.010 (3)	−0.0039 (18)	0.009 (2)
O32	0.0285 (13)	0.029 (3)	0.040 (3)	−0.0141 (19)	−0.0021 (17)	0.0005 (19)
C5	0.013 (3)	0.0270 (18)	0.037 (3)	−0.012 (3)	0.001 (2)	0.011 (3)
C6	0.033 (3)	0.0180 (15)	0.0315 (16)	−0.002 (2)	−0.0128 (19)	0.0007 (12)
C1	0.0289 (15)	0.0196 (16)	0.0222 (19)	−0.0039 (13)	0.0000 (14)	0.0016 (17)
C2	0.008 (5)	0.0230 (19)	0.0321 (16)	−0.011 (4)	0.0036 (16)	−0.0013 (12)
C3	0.017 (3)	0.0249 (18)	0.049 (4)	−0.006 (2)	−0.008 (3)	−0.004 (3)
C4	0.0279 (18)	0.0281 (19)	0.042 (5)	−0.0003 (16)	0.001 (3)	0.008 (4)
C12	0.014 (2)	0.0207 (19)	0.036 (3)	0.0019 (16)	0.0043 (15)	0.0121 (18)
C13	0.0324 (18)	0.028 (2)	0.0122 (16)	−0.0036 (16)	−0.0053 (15)	−0.0027 (15)
C14	0.029 (3)	0.0229 (19)	0.0102 (16)	−0.012 (3)	−0.002 (3)	0.0026 (14)
O15'	0.019 (3)	0.027 (3)	0.0207 (17)	−0.0050 (19)	−0.0049 (17)	0.0026 (15)
O18'	0.0330 (17)	0.0225 (15)	0.036 (2)	−0.0023 (13)	0.0025 (16)	−0.0022 (16)
O20'	0.025 (2)	0.063 (2)	0.039 (2)	−0.010 (3)	−0.0039 (18)	0.009 (2)
O32'	0.0285 (13)	0.029 (3)	0.040 (3)	−0.0141 (19)	−0.0021 (17)	0.0005 (19)
C5'	0.013 (3)	0.0270 (18)	0.037 (3)	−0.012 (3)	0.001 (2)	0.011 (3)
C6'	0.033 (3)	0.0180 (15)	0.0315 (16)	−0.002 (2)	−0.0128 (19)	0.0007 (12)
C1'	0.0289 (15)	0.0196 (16)	0.0222 (19)	−0.0039 (13)	0.0000 (14)	0.0016 (17)
C2'	0.008 (5)	0.0230 (19)	0.0321 (16)	−0.011 (4)	0.0036 (16)	−0.0013 (12)
C3'	0.017 (3)	0.0249 (18)	0.049 (4)	−0.006 (2)	−0.008 (3)	−0.004 (3)
C4'	0.0279 (18)	0.0281 (19)	0.042 (5)	−0.0003 (16)	0.001 (3)	0.008 (4)
C12'	0.014 (2)	0.0207 (19)	0.036 (3)	0.0019 (16)	0.0043 (15)	0.0121 (18)
C13'	0.0324 (18)	0.028 (2)	0.0122 (16)	−0.0036 (16)	−0.0053 (15)	−0.0027 (15)
C14'	0.029 (3)	0.0229 (19)	0.0102 (16)	−0.012 (3)	−0.002 (3)	0.0026 (14)

### Geometric parameters (Å, º)

**Table:**

O7—C1	1.361 (5)	C31—H31A	0.9800
O7—C1'	1.381 (6)	C31—H31B	0.9800
O7—C8	1.434 (4)	C31—H31C	0.9800
O9—C2'	1.318 (6)	C33—H33A	0.9800
O9—C10	1.357 (4)	C33—H33B	0.9800
O9—C2	1.468 (5)	C33—H33C	0.9800
O11—C10	1.191 (4)	C34—H34A	0.9800
O22—C21	1.333 (3)	C34—H34B	0.9800
O22—C23	1.485 (4)	C34—H34C	0.9800
O26—C25	1.316 (5)	C25—O32'	1.247 (6)
O26—C27	1.489 (4)	C25—O32	1.258 (6)
O29—C21	1.202 (4)	O15—C14	1.494 (8)
C8—H8A	0.9800	O18—H18	0.8400
C8—H8B	0.9800	O20—C14	1.146 (9)
C8—H8C	0.9800	C5—C6	1.3900
C10—C19	1.495 (4)	C5—C4	1.3900
C19—H19A	0.9800	C5—C12	1.608 (7)
C19—H19B	0.9800	C6—C1	1.3900
C19—H19C	0.9800	C6—H6	0.9500
C16—O15'	1.441 (5)	C1—C2	1.3900
C16—O15	1.457 (5)	C2—C3	1.3900
C16—C21	1.519 (4)	C3—C4	1.3900
C16—C17	1.525 (4)	C3—H3	0.9500
C16—H16	1.0000	C4—H4	0.9500
C16—H16'	1.0000	C12—C13	1.313 (7)
C17—O18'	1.450 (5)	C12—H12	0.9500
C17—O18	1.460 (5)	C13—C14	1.495 (7)
C17—C25	1.530 (6)	C13—H13	0.9500
C17—H17	1.0000	O15'—C14'	1.447 (7)
C17—H17'	1.0000	O18'—H18'	0.8400
C23—C31	1.517 (5)	O20'—C14'	1.203 (9)
C23—C24	1.523 (4)	C5'—C6'	1.3900
C23—C30	1.526 (4)	C5'—C4'	1.3900
C24—H24A	0.9800	C5'—C12'	1.497 (6)
C24—H24B	0.9800	C6'—C1'	1.3900
C24—H24C	0.9800	C6'—H6'	0.9500
C27—C28	1.508 (8)	C1'—C2'	1.3900
C27—C34	1.517 (5)	C2'—C3'	1.3900
C27—C33	1.524 (6)	C3'—C4'	1.3900
C28—H28A	0.9800	C3'—H3'	0.9500
C28—H28B	0.9800	C4'—H4'	0.9500
C28—H28C	0.9800	C12'—C13'	1.333 (7)
C30—H30A	0.9800	C12'—H12'	0.9500
C30—H30B	0.9800	C13'—C14'	1.468 (7)
C30—H30C	0.9800	C13'—H13'	0.9500
			
C1—O7—C8	119.0 (6)	C23—C31—H31B	109.5
C1'—O7—C8	116.3 (5)	H31A—C31—H31B	109.5
C2'—O9—C10	110.8 (9)	C23—C31—H31C	109.5
C10—O9—C2	119.4 (8)	H31A—C31—H31C	109.5
C21—O22—C23	121.9 (2)	H31B—C31—H31C	109.5
C25—O26—C27	122.2 (3)	C27—C33—H33A	109.5
O7—C8—H8A	109.5	C27—C33—H33B	109.5
O7—C8—H8B	109.5	H33A—C33—H33B	109.5
H8A—C8—H8B	109.5	C27—C33—H33C	109.5
O7—C8—H8C	109.5	H33A—C33—H33C	109.5
H8A—C8—H8C	109.5	H33B—C33—H33C	109.5
H8B—C8—H8C	109.5	C27—C34—H34A	109.5
O11—C10—O9	122.8 (3)	C27—C34—H34B	109.5
O11—C10—C19	125.6 (3)	H34A—C34—H34B	109.5
O9—C10—C19	111.6 (3)	C27—C34—H34C	109.5
C10—C19—H19A	109.5	H34A—C34—H34C	109.5
C10—C19—H19B	109.5	H34B—C34—H34C	109.5
H19A—C19—H19B	109.5	O32'—C25—O26	114.1 (4)
C10—C19—H19C	109.5	O32—C25—O26	141.5 (4)
H19A—C19—H19C	109.5	O32'—C25—C17	135.1 (5)
H19B—C19—H19C	109.5	O32—C25—C17	107.3 (4)
O15'—C16—C21	105.1 (3)	O26—C25—C17	110.7 (3)
O15—C16—C21	113.7 (4)	C16—O15—C14	112.4 (4)
O15'—C16—C17	100.0 (3)	C17—O18—H18	109.5
O15—C16—C17	113.8 (3)	C6—C5—C4	120.0
C21—C16—C17	109.6 (3)	C6—C5—C12	116.8 (6)
O15'—C16—H16	128.5	C4—C5—C12	123.2 (6)
O15—C16—H16	106.4	C5—C6—C1	120.0
C21—C16—H16	106.4	C5—C6—H6	120.0
C17—C16—H16	106.4	C1—C6—H6	120.0
O15'—C16—H16'	113.8	O7—C1—C2	121.8 (8)
O15—C16—H16'	91.7	O7—C1—C6	117.9 (7)
C21—C16—H16'	113.5	C2—C1—C6	120.0
C17—C16—H16'	113.7	C1—C2—C3	120.0
O18'—C17—C16	106.2 (3)	C1—C2—O9	111.4 (7)
O18—C17—C16	112.6 (3)	C3—C2—O9	127.9 (8)
O18'—C17—C25	96.5 (3)	C4—C3—C2	120.0
O18—C17—C25	119.7 (3)	C4—C3—H3	120.0
C16—C17—C25	110.3 (3)	C2—C3—H3	120.0
O18'—C17—H17	134.3	C3—C4—C5	120.0
O18—C17—H17	104.2	C3—C4—H4	120.0
C16—C17—H17	104.2	C5—C4—H4	120.0
C25—C17—H17	104.2	C13—C12—C5	122.9 (6)
O18'—C17—H17'	113.6	C13—C12—H12	118.6
O18—C17—H17'	83.4	C5—C12—H12	118.6
C16—C17—H17'	114.5	C12—C13—C14	127.0 (6)
C25—C17—H17'	114.2	C12—C13—H13	116.5
O29—C21—O22	126.6 (3)	C14—C13—H13	116.5
O29—C21—C16	125.7 (3)	O20—C14—O15	124.5 (6)
O22—C21—C16	107.7 (2)	O20—C14—C13	127.8 (6)
O22—C23—C31	108.8 (3)	O15—C14—C13	107.5 (6)
O22—C23—C24	110.6 (2)	C16—O15'—C14'	107.0 (4)
C31—C23—C24	112.7 (3)	C17—O18'—H18'	109.5
O22—C23—C30	102.5 (2)	C6'—C5'—C4'	120.0
C31—C23—C30	111.0 (3)	C6'—C5'—C12'	117.2 (5)
C24—C23—C30	110.7 (3)	C4'—C5'—C12'	122.2 (6)
C23—C24—H24A	109.5	C1'—C6'—C5'	120.0
C23—C24—H24B	109.5	C1'—C6'—H6'	120.0
H24A—C24—H24B	109.5	C5'—C6'—H6'	120.0
C23—C24—H24C	109.5	O7—C1'—C6'	131.5 (8)
H24A—C24—H24C	109.5	O7—C1'—C2'	107.8 (8)
H24B—C24—H24C	109.5	C6'—C1'—C2'	120.0
O26—C27—C28	102.7 (3)	O9—C2'—C3'	110.2 (8)
O26—C27—C34	108.9 (3)	O9—C2'—C1'	128.8 (8)
C28—C27—C34	111.5 (4)	C3'—C2'—C1'	120.0
O26—C27—C33	108.3 (4)	C2'—C3'—C4'	120.0
C28—C27—C33	111.8 (4)	C2'—C3'—H3'	120.0
C34—C27—C33	113.0 (4)	C4'—C3'—H3'	120.0
C27—C28—H28A	109.5	C3'—C4'—C5'	120.0
C27—C28—H28B	109.5	C3'—C4'—H4'	120.0
H28A—C28—H28B	109.5	C5'—C4'—H4'	120.0
C27—C28—H28C	109.5	C13'—C12'—C5'	128.8 (5)
H28A—C28—H28C	109.5	C13'—C12'—H12'	115.6
H28B—C28—H28C	109.5	C5'—C12'—H12'	115.6
C23—C30—H30A	109.5	C12'—C13'—C14'	117.6 (6)
C23—C30—H30B	109.5	C12'—C13'—H13'	121.2
H30A—C30—H30B	109.5	C14'—C13'—H13'	121.2
C23—C30—H30C	109.5	O20'—C14'—O15'	125.5 (6)
H30A—C30—H30C	109.5	O20'—C14'—C13'	128.0 (6)
H30B—C30—H30C	109.5	O15'—C14'—C13'	106.4 (5)
C23—C31—H31A	109.5		
			
C2'—O9—C10—O11	−9.0 (7)	C6—C1—C2—C3	0.0
C2—O9—C10—O11	−2.9 (7)	O7—C1—C2—O9	−3.1 (9)
C2'—O9—C10—C19	170.5 (6)	C6—C1—C2—O9	171.6 (14)
C2—O9—C10—C19	176.6 (6)	C2'—O9—C2—C1	−46 (8)
O15'—C16—C17—O18'	−43.9 (4)	C10—O9—C2—C1	−80.4 (7)
O15—C16—C17—O18'	−62.3 (5)	C2'—O9—C2—C3	124 (9)
C21—C16—C17—O18'	66.2 (4)	C10—O9—C2—C3	90.4 (12)
O15'—C16—C17—O18	−77.0 (4)	C1—C2—C3—C4	0.0
O15—C16—C17—O18	−95.5 (5)	O9—C2—C3—C4	−170.1 (16)
C21—C16—C17—O18	33.1 (4)	C2—C3—C4—C5	0.0
O15'—C16—C17—C25	59.5 (4)	C6—C5—C4—C3	0.0
O15—C16—C17—C25	41.1 (4)	C12—C5—C4—C3	179.9 (8)
C21—C16—C17—C25	169.6 (2)	C6—C5—C12—C13	−151.1 (8)
C23—O22—C21—O29	6.2 (5)	C4—C5—C12—C13	29.1 (9)
C23—O22—C21—C16	−173.2 (3)	C5—C12—C13—C14	−176.5 (7)
O15'—C16—C21—O29	−6.2 (5)	C16—O15—C14—O20	−11.7 (10)
O15—C16—C21—O29	15.7 (5)	C16—O15—C14—C13	172.4 (5)
C17—C16—C21—O29	−112.9 (4)	C12—C13—C14—O20	−169.6 (8)
O15'—C16—C21—O22	173.3 (3)	C12—C13—C14—O15	6.0 (9)
O15—C16—C21—O22	−164.8 (3)	O15—C16—O15'—C14'	−14.2 (10)
C17—C16—C21—O22	66.6 (3)	C21—C16—O15'—C14'	101.9 (5)
C21—O22—C23—C31	64.2 (3)	C17—C16—O15'—C14'	−144.5 (5)
C21—O22—C23—C24	−60.1 (4)	C4'—C5'—C6'—C1'	0.0
C21—O22—C23—C30	−178.2 (3)	C12'—C5'—C6'—C1'	−171.2 (8)
C25—O26—C27—C28	179.7 (3)	C1—O7—C1'—C6'	114 (16)
C25—O26—C27—C34	−62.0 (5)	C8—O7—C1'—C6'	−5.3 (18)
C25—O26—C27—C33	61.3 (5)	C1—O7—C1'—C2'	−56 (14)
C27—O26—C25—O32'	−3.6 (5)	C8—O7—C1'—C2'	−175.5 (4)
C27—O26—C25—O32	8.4 (8)	C5'—C6'—C1'—O7	−169.2 (19)
C27—O26—C25—C17	179.3 (3)	C5'—C6'—C1'—C2'	0.0
O18'—C17—C25—O32'	−18.1 (7)	C10—O9—C2'—C3'	105.5 (8)
O18—C17—C25—O32'	5.0 (7)	C2—O9—C2'—C3'	−43 (8)
C16—C17—C25—O32'	−128.0 (6)	C10—O9—C2'—C1'	−86.2 (10)
O18'—C17—C25—O32	−27.7 (5)	C2—O9—C2'—C1'	125 (9)
O18—C17—C25—O32	−4.6 (6)	O7—C1'—C2'—O9	4.3 (9)
C16—C17—C25—O32	−137.6 (4)	C6'—C1'—C2'—O9	−167.3 (17)
O18'—C17—C25—O26	158.2 (3)	O7—C1'—C2'—C3'	171.5 (16)
O18—C17—C25—O26	−178.7 (3)	C6'—C1'—C2'—C3'	0.0
C16—C17—C25—O26	48.3 (4)	O9—C2'—C3'—C4'	169.5 (14)
O15'—C16—O15—C14	155.9 (17)	C1'—C2'—C3'—C4'	0.0
C21—C16—O15—C14	84.7 (6)	C2'—C3'—C4'—C5'	0.0
C17—C16—O15—C14	−148.9 (5)	C6'—C5'—C4'—C3'	0.0
C4—C5—C6—C1	0.0	C12'—C5'—C4'—C3'	170.8 (8)
C12—C5—C6—C1	−179.9 (7)	C6'—C5'—C12'—C13'	12.2 (11)
C1'—O7—C1—C2	125 (15)	C4'—C5'—C12'—C13'	−158.8 (7)
C8—O7—C1—C2	−171.5 (5)	C5'—C12'—C13'—C14'	179.1 (6)
C1'—O7—C1—C6	−49 (14)	C16—O15'—C14'—O20'	−13.8 (10)
C8—O7—C1—C6	13.7 (15)	C16—O15'—C14'—C13'	168.8 (5)
C5—C6—C1—O7	174.9 (17)	C12'—C13'—C14'—O20'	16.2 (11)
C5—C6—C1—C2	0.0	C12'—C13'—C14'—O15'	−166.5 (6)
O7—C1—C2—C3	−174.7 (18)		

### Hydrogen-bond geometry (Å, º)

**Table:**

*D*—H···*A*	*D*—H	H···*A*	*D*···*A*	*D*—H···*A*
O18—H18···O32	0.84	2.21	2.631 (8)	111
O18—H18···O22^i^	0.84	2.57	3.185 (5)	131

Symmetry code: (i) *x*−1, *y*, *z*.
